# Mental health, substance use and viral suppression in adolescents receiving ART at a paediatric HIV clinic in South Africa

**DOI:** 10.1002/jia2.25644

**Published:** 2020-12-07

**Authors:** Andreas D Haas, Karl‐Günter Technau, Shenaaz Pahad, Kate Braithwaite, Mampho Madzivhandila, Gillian Sorour, Shobna Sawry, Nicola Maxwell, Per von Groote, Mpho Tlali, Mary‐Ann Davies, Matthias Egger, for the IeDEA Southern Africa Collaboration

**Affiliations:** ^1^ Institute of Social & Preventive Medicine University of Bern Bern Switzerland; ^2^ Empilweni Services and Research Unit Department of Paediatrics & Child Health Rahima Moosa Mother and Child Hospital Faculty of Health Sciences University of the Witwatersrand Johannesburg South Africa; ^3^ Wits Reproductive Health and HIV Institute Faculty of Health Sciences University of the Witwatersrand Johannesburg South Africa; ^4^ Centre for Infectious Disease Epidemiology and Research University of Cape Town Cape Town South Africa; ^5^ Population Health Sciences Bristol Medical School University of Bristol Bristol United Kingdom

**Keywords:** adolescents, HIV, antiretroviral therapy, viral suppression, mental disorders, South Africa

## Abstract

**Introduction:**

Mental health problems are prevalent in adolescents living with HIV (ALHIV), often remain untreated, and may negatively affect antiretroviral therapy (ART) adherence and viral suppression. We implemented routine mental health screening at a paediatric ART clinic to improve the identification and management of mental health problems in ALHIV. In this report, we examine screening outcomes, associated patient characteristics and the odds of unsuppressed viral load in ALHIV screening positive for mental disorders.

**Methods:**

Adolescents aged 10 to 19 years attending Rahima Moosa Hospital in Johannesburg, South Africa between February 1, 2018, and January 1, 2020, were offered mental health screening at each routine HIV care visit. The screening included four pre‐screening questions followed by full screening (conditional on positive pre‐screening) for depression (Patient Health Questionnaire‐9 [PHQ‐9]), suicide (Adolescent Innovations Project [AIP]‐handbook), anxiety (Generalized Anxiety Disorder‐7 [GAD‐7]), post‐traumatic stress disorder (PTSD) (Primary Care PTSD Screen [PC‐PTSD‐5]) and substance use (CAGE Adapted to Include Drugs [CAGE‐AID]). We assessed screening outcomes and calculated adjusted odds ratios for associations between positive screening tests at the first screen and unsuppressed viral load (>400 copies/mL) at the measurement taken closest to the date of screening, within hundred days before and one day after screening.

**Results:**

Out of 1203 adolescents who attended the clinic, 1088 (90.4%) were pre‐screened of whom 381 (35.0%) underwent full screening, 48 (4.4%) screened positive for depression (PHQ‐9 ≥10), 29 (2.8%) for suicidal concern, 24 (2.2%) for anxiety (GAD‐7 ≥10), 38 (3.2%) for PTSD (PC‐PTSD‐5 ≥3), 18 (1.7%) for substance use (CAGE‐AID ≥2) and 97 (8.9%) for any of these conditions. Positive screening for depression (aOR 2.39, 95% CI 1.02 to 5.62), PTSD (aOR 3.18, 95% CI 1.11 to 9.07), substance use (aOR 7.13, 95% CI 1.60 to 31.86), or any condition (aOR 2.17, 95% CI 1.17 to 4.02) were strongly associated with unsuppressed viral load.

**Conclusions:**

ALHIV affected by mental health problems have increased rates of unsuppressed viral load and need specific clinical attention. The integration of routine mental health screening in paediatric ART programmes is a feasible approach for identifying and referring adolescents with mental health and adherence problems to counselling and psychosocial support services and if needed to psychiatric care.

## 1 INTRODUCTION

In 2018, there were an estimated 1.6 million adolescents (aged 10 to 19 years) living with HIV (ALHIV) globally [1]. The majority of ALHIV live in sub‐Saharan Africa, and about 20% in South Africa [1]. ALHIV are a heterogeneous group comprising of those who acquired HIV perinatally and survived into adolescence and those who acquired HIV through sexual transmission [2, 3, 4]. In South Africa, the majority of ALHIV enrolled in HIV care, acquired HIV perinatally [5].

For a variety of reasons, ALHIV are at risk of developing mental health complications, including depression, anxiety and post‐traumatic stress disorder (PTSD). HIV infection may have a direct effect on the brain, which can lead to neurocognitive decline, behavioural disturbance, mood disorders and psychosis [6]. Furthermore, certain antiretroviral drugs used to treat HIV may have neuropsychiatric side effects [7]. In addition to these biological mechanisms that link HIV and mental illness, several social and structural factors contribute to the poor mental health of ALHIV. Many ALHIV have lost their biological parents and are raised in extended families, or orphanages [8, 9]. Caregivers of orphans might be overburdened by the responsibility to care for, and economically support an additional child or children [9]. Orphans often lack social and material support, feel unsafe at home, are uncertain about their future and suffer from bereavement following the loss of their biological parents [10, 11, 12, 13, 14, 15]. ALHIV frequently experience verbal abuse, bullying, violence, stigma and discrimination [14, 16, 17, 18, 19, 20, 21], often internalize stigma and suffer from self‐blame [13, 22, 23]. Lastly, ALHIV have to cope with issues related to the disclosure of their HIV status and the burden of lifelong HIV treatment [12, 24, 25].

Mental health and substance use problems including depression, anxiety, alcohol abuse and illicit drug use can adversely affect adherence to antiretroviral therapy (ART) [26, 27, 28, 29]. Such problems may lead to poor HIV treatment outcomes including unsuppressed viral load, loss to follow‐up and premature mortality [30]. However, evidence on associations between mental health and markers of HIV treatment response among ALHIV is inconclusive. Some studies reported associations between mental health problems and low CD4 percentage, higher rates of unsuppressed HIV viral load and HIV disease progression [10, 31, 32, 33, 34]. Still, other studies found no evidence of such associations [10, 35, 36].

In low‐ and middle‐income countries capacity to diagnose and manage mental disorders is limited [37, 38]. The South African national treatment guidelines recommend routine mental health screening as part of adherence support [39]. Still, the implementation of mental health services in the South African healthcare system is inconsistent [40, 41]. Routine mental health screening is rarely done in public‐sector ART programmes in South Africa, and the vast majority of ART patients affected by mental health problems remain undiagnosed and untreated [42].

We implemented routine mental health screening at a large paediatric HIV clinic in Johannesburg, South Africa to improve the identification and management of ALHIV affected by mental health problems. In this report, we describe mental health screening outcomes of ALHIV screened between February 1, 2018, and January 1, 2020, and examine associated patient characteristics, and the increase in the odds of unsuppressed viral load in ALHIV screening positive for a mental disorder.

## 2 METHODS

### 2.1 Study design

We followed a cohort of HIV‐positive adolescents who enrolled in HIV care at the Rahima Moosa Mother and Child Hospital in Johannesburg, South Africa. The clinic participates in the International epidemiology Databases to Evaluate AIDS in Southern Africa (IeDEA‐SA) and regularly transfers de‐identified routine clinical data of HIV‐positive mothers, children and adolescents to the IeDEA‐SA data centres at the University of Cape Town and the University of Bern [43].

### 2.2 Setting and participants

Rahima Moosa Mother and Child Hospital is an academic, public hospital. The clinic is the second largest ART clinic for the treatment of children and ALHIV in Gauteng province. We included ALHIV, aged 10 to 19 years, who received ART at Rahima Moosa Mother and Child Hospital and participated at least once in mental health screenings during the study period from February 1, 2018, to January 1, 2020.

### 2.3 Screening procedure

We offered adolescents aged 10 to 19 years old routine mental health screening at every three monthly follow‐up visit to the clinic. The screening consisted of a pre‐screen for depression, anxiety, PTSD and substance use, followed by full screen among adolescents with a positive pre‐screen (Table 1). The full screen included the Patient Health Questionnaire (PHQ‐9), the Generalized Anxiety Disorder 7 item (GAD‐7) scale, the Primary Care PTSD Screen for DSM‐5 (PC‐PTSD‐5) and the CAGE Adapted to Include Drugs (CAGE‐AID) questionnaire (Table 1) [44, 45, 46, 47]. Adolescents who screened positive for depression in the pre‐screening also received a full screening for suicidality using the three‐item screening tool from the Adolescent Innovations Project (AIP) working with Adolescents living with HIV handbook [48]. In addition to mental health questions, screenings included six basic questions on food insecurity, assault, household conflicts and socioeconomic situation. A clinical psychologist trained personnel in administering the screening tools. The implementation of the screening programme was jointly overseen by a senior doctor and a social worker with experience in working with ALHIV with mental health problems. Counsellors (68.2 %, 3044/4461) and nurses (25.4%, 1131) conducted most screens. Few screens (0.7%, 33) were conducted by doctors, social workers, or psychologists and the information on the screener was missing for 5.7% (253) of the screens. Adolescents who screened positive in a full screen were referred and assessed by a senior doctor and social worker within the clinic and directed towards conclusive management, which included referrals for counselling by onsite psychologists, referral to psychiatric services, peer‐support, family meetings and adherence counselling.

**Table 1:** Mental health and substance use screening model

Condition	Pre‐screen			Full screen	
	Question	Answers (points)	Positive screen	Tool	Positive screen
Depression	How often do you feel sad?	Every day (4) Most of the time: five to six days a week (3) Sometimes: three to four days a week (2) Not much: one to two days a week (1) Never (0)	2 or higher	Screening for depression using the Patient Health Questionnaire (PHQ‐9) [44]	10 or higher
Suicidality			2 or higher on depression pre‐screen	Screening for suicidality using the Adolescent Innovations Project (AIP) working with Adolescents living with HIV handbook [48]	Past or current thoughts of self‐harm/suicide or previous suicide attempt
Anxiety	How often do you feel worried?	Every day (4) Most of the time: five to six days a week (3) Sometimes: three to four days a week (2) Not much: one to two days a week (1) Never (0)	2 or higher	Screening for anxiety using the Generalized Anxiety Disorder 7‐item (GAD‐7) scale [45]	10 or higher
Post‐traumatic stress disorder (PTSD)	Have you ever/since your last visit had a bad experience where you were scared that you or someone you love would be seriously hurt or killed?	Yes No	Yes	Screening for PTSD using the Primary Care PTSD Screen for DSM‐5 (PC‐PTSD‐5) [46]	3 or higher
Substance use	Do you drink alcohol or use drugs?	Yes No	Yes	Screening for substance use using the CAGE Adapted to Include Drugs (CAGE‐AID) scale [47]	2 or higher

Screening tools have been validated in various settings and populations including in low‐ and middle‐income settings and adolescents and showed mostly good (area under the curve [AUC] >0.8) or acceptable (AUC>0.7) diagnostic accuracy [44, 59]. However, none of the tools has been validated in South African ALHIV.

### 2.4 Measures

Adolescents who reported past or current thoughts of self‐harm or suicide or reported a previous suicide attempt screened positive in the full screening for suicidality. A PHQ‐9 or GAD‐7 score of 10 or higher was considered a positive full screen for depression and anxiety, respectively. For PTSD, a PC‐PTSD‐5 score of 3 or higher and for substance use a CAGE‐AID score of 2 or higher were considered positive full screens. The final screening outcome was classified as positive if an adolescent ever screened positive in any full screen. It was classified as negative if an adolescent always screened negative in pre‐screens or positive in pre‐screens and negative in full screens. We defined the CD4 count at the start of the study as the value closest to the start of the study on February 1, 2018, within one year prior, and one year after that date. The window for CD4 at ART initiation was six months prior and one month after ART initiation. We defined unsuppressed viral load as one viral load above 400 copies/mL. We defined viral load at screening as the measurement taken closest to the date of screening, within hundred days before and one day after the screening.

### 2.5 Statistical analysis

We used descriptive statistics to examine characteristics of adolescents stratified by the final screening outcome. We calculated percentages of adolescents who screened positive, at the first screen, or ever, in pre‐ and full screens for depression, suicidality, anxiety, PTSD, substance use or any condition. Full screens were done conditionally on a positive pre‐screen. For proportion, the denominator was the total population pre‐screened. We calculated the mean and standard deviation of the PHQ‐9, GAD‐7, PC‐PTSD‐5 and CAGE‐AID scores of adolescents who had a positive full screen for the particular condition. We estimated the percentage of adolescents who had a positive full screen for two conditions for each possible pair of conditions and plotted the results in a heat map.

We calculated unadjusted odds ratios (OR) and adjusted odds ratios (aOR) with 95% confidence intervals (CI) for patient characteristics associated with ever screening positive in full screens for depression, suicidal concern, anxiety, PTSD and alcohol/substance abuse using logistic regression. We considered a priori hypothesized predictors of mental health and substance use problems including sociodemographic and clinical characteristics, and factors describing the life circumstances of adolescents in univariable analysis. Variables associated with a positive screen at a significance level of α < 0.2 in univariable analysis were considered in multivariable analysis. Variables that were not significant at α < 0.05 in multivariable analysis were eliminated from the final model [60].

We calculated ORs and aORs with 95% CIs for associations between positive full screens at the first screen and unsuppressed viral load at screening using logistic regression. Viral load testing was routinely performed annually. Hence, viral load testing was not necessarily performed on the day of mental health screening. We restricted the analysis of associations between positive full screens and unsuppressed viral load to adolescents who had a viral load test performed within hundred days before and one day after the screening. We did not consider viral load tests performed later than one day after screening because intervention following a positive mental health screen might influence viral suppression, and therefore, might distort associations between screening outcomes and unsuppressed viral load. In multivariable analysis, we adjusted for gender, age at screening (10 to 12, 13 to 15, or 16 to 19 years), regimen at screening (non‐nucleoside reverse transcriptase inhibitor [NNRTI]‐based, protease inhibitor [PI]‐based, or other), age at ART initiation (<2, 2 to 4, 5 to 9, or 10 years or older), CD4 cell count at ART initiation (<100, 100 to 199, 200 to 349, 350 to 499, ≥500 cells/µL or unknown) and regimen at ART initiation (NNRTI‐based, PI‐based, or other). These potential confounders were selected a priori. In a sensitivity analysis, we assessed associations of positive mental and substance use screens and unsuppressed viral load at a threshold of above 1000 copies/mL. Statistical analysis was done in Stata (Version 16, Stata Corporation, College Station, TX).

### 2.6 Ethical considerations

The clinic has institutional ethical approval for the contribution of data to IeDEA. The Human Research Ethics Committee of the University of Cape Town, South Africa and the Cantonal Ethics Committee, Bern, Switzerland granted permission for analysis of this database. Institutional review boards have granted waivers of informed consent as the analyses use only de‐identified data that are collected as part of routine patient care.

## 3 RESULTS

### 3.1 Screening coverage

During the study period, 1203 ART patients aged 10 to 19 visited the clinic, of whom 1088 (90.4%) were screened (i.e. at least pre‐screened) and included in our study, of whom 381 (35.0%) underwent full screening for at least one condition. The reasons for omitting screening in 115 adolescents (9.6%) were not documented. On average, adolescents participating in screenings were screened eight times (IQR 6 to 9) over a median duration of 518 days (IQR 420 to 576) between first and last screening.

### 3.2 Characteristics of adolescents screened for mental health and substance use problems

Tables 2 and 3 show demographic, clinical and other characteristics adolescents screened. Half of the 1088 adolescents screened were male. At the beginning of the study period, the median age of the study population was 13 years (IQR 10 to 15), adolescents were receiving ART for a median duration of 9 years (IQR 6 to 11 years). At ART initiation, the median age of adolescents was 3 years (IQR 1 to 7), median CD4 cell count was 496 cells/µL (IQR 262 to 853; Table 2). Few adolescents (5.9%) reported that they had experienced food insecurity, 15.3% reported that they had experienced physical violence and 14.1% reported a current or past conflict at home (Table 3).

**Table 2:** Demographic and clinical characteristics of adolescents screened for mental health and substance use problems in a paediatric HIV clinic in South Africa

	Final screening outcome^a^, n (%)		
	Negative	Positive	Total, n (%)
	N = 991	N = 97	N = 1088
Characteristics at the start of the study^b^			
Gender			
Male	498 (50.3)	51 (52.6)	549 (50.5)
Female	493 (49.7)	46 (47.4)	539 (49.5)
Years on ART			
0 to 5	193 (19.5)	12 (12.4)	205 (18.8)
6 to 10	527 (53.2)	45 (46.4)	572 (52.6)
>10	271 (27.3)	40 (41.2)	311 (28.6)
Median (IQR)	9 (6 to 11)	10 (7 to 12)	9 (6 to 11)
Regimen			
NNRTI‐based	640 (64.6)	59 (60.8)	699 (64.2)
PI‐based	336 (33.9)	38 (39.2)	374 (34.4)
Other	15 (1.5)	0 (0.0)	15 (1.4)
Age in years			
9 to 12	522 (52.7)	16 (16.5)	538 (49.4)
13 to 15	304 (30.7)	25 (25.8)	329 (30.2)
16 to 19	165 (16.6)	56 (57.7)	221 (20.3)
Median (IQR)	12 (10 to 14)	16 (14 to 17)	13 (10 to 15)
CD4 count in cells/µL			
<350	72 (7.3)	13 (13.4)	85 (7.8)
350 to 499	104 (10.5)	18 (18.6)	122 (11.2)
500 to 749	275 (27.7)	27 (27.8)	302 (27.8)
750+	469 (47.3)	33 (34.0)	502 (46.1)
Unknown	71 (7.2)	6 (6.2)	77 (7.1)
Median (IQR)	766 (545 to 990)	671 (397 to 840)	747 (537 to 982
Characteristics at ART initiation			
Regimen			
NNRTI‐based	562 (56.7)	73 (75.3)	635 (58.4)
PI‐based	379 (38.2)	23 (23.7)	402 (36.9)
Other	50 (5.0)	1 (1.0)	51 (4.7)
Age in years			
<2	386 (39.0)	15 (15.5)	401 (36.9)
2 to 4	225 (22.7)	29 (29.9)	254 (23.3)
5 to 9	279 (28.2)	40 (41.2)	319 (29.3)
10 to 19	101 (10.2)	13 (13.4)	114 (10.5)
Median (IQR)	3 (1 to 6)	5 (3 to 8)	3 (1 to 7)
CD4 count in cells/µL			
<100	72 (7.3)	9 (9.3)	81 (7.4)
100 to 199	38 (3.8)	7 (7.2)	45 (4.1)
200 to 349	92 (9.3)	11 (11.3)	103 (9.5)
350 to 499	92 (9.3)	9 (9.3)	101 (9.3)
500+	302 (30.5)	24 (24.7)	326 (30.0)
Unknown	395 (39.9)	37 (38.1)	432 (39.7)
Median (IQR)	505 (271 to 873)	410 (186 to 648)	496 (262 to 853)

Data are n (%) unless otherwise specified. ART, antiretroviral therapy; NNRTI, non‐nucleoside reverse transcriptase inhibitors; PI, protease inhibitors; IQR, interquartile range.

^a^ The final screening outcome was classified as positive if an adolescent ever screened positive in any full screen or else (i.e. always negative pre‐screen or positive pre‐ and negative full screen) as negative

^b^ beginning of the study period was February 01, 2018.

**Table 3:** Life circumstances of adolescents screened for mental health and substance use problems at a paediatric HIV clinic in South Africa

	Final screening outcome^a^, n (%)		
	Negative	Positive	Total, n (%)
	N = 991	N = 97	N = 1088
Experienced food insecurity			
Never	936 (94.5)	87 (89.7)	1023 (94.0)
Currently or in the past	54 (5.4)	10 (10.3)	64 (5.9)
Unknown/refused/missing	1 (0.1)	0 (0.0)	1 (0.1)
Experienced physical violence			
Never	844 (85.2)	75 (77.3)	919 (84.5)
Currently or in the past	146 (14.7)	21 (21.6)	167 (15.3)
Unknown/refused/missing	1 (0.1)	1 (1.0)	2 (0.2)
Conflict in household			
Never	863 (87.1)	71 (73.2)	934 (85.8)
Currently or in the past	127 (12.8)	26 (26.8)	153 (14.1)
Unknown/refused/missing	1 (0.1)	0 (0.0)	1 (0.1)
Employment of household member			
Never	35 (3.5)	2 (2.1)	37 (3.4)
Currently or in the past	955 (96.4)	95 (97.9)	1050 (96.5)
Unknown/refused/missing	1 (0.1)	0 (0.0)	1 (0.1)
Household grant			
Never	190 (19.2)	24 (24.7)	214 (19.7)
Currently or in the past	745 (75.2)	72 (74.2)	817 (75.1)
Unknown/refused/missing	56 (5.7)	1 (1.0)	57 (5.2)

Questions regarding life circumstances were asked after each mental health and substance use screening. Data are numbers and percentages of adolescents who reported the five life circumstances at any time during the study duration.

^a^ The final screening outcome was classified as positive if an adolescent ever screened positive in any full screen or else (i.e. always negative pre‐screen or positive pre‐ and negative full screen) as negative.

### 3.3 Screening outcomes and management of adolescents with positive screening outcomes

Table 4 shows the proportions of adolescents who screened positive in pre‐ and full screens for depression, suicidality, anxiety, PTSD, substance use or any of these conditions. Out of all adolescents who were pre‐screened, 6.9% had a positive full screen for any condition at the first screen: 3.6% for depression, 2.2% for suicidal concern, 1.9% for anxiety, 2.2% for PTSD and 0.9% for alcohol/substance abuse. The prevalence of positive full screens increased with age. In repeated screening, few additional adolescents screened positive in full screens. In total, 8.9% of pre‐screened adolescents ever screened positive in a full screen for any condition: 4.4% for depression, 2.7% for suicidal concern, 2.2% for anxiety, 3.5% for PTSD and 1.7% for alcohol/substance abuse. Out of the 97 adolescents who screened positive in full screens, 97 (100.0%) were seen and assessed by a senior doctor and a social worker, 16 (16.5%) were referred to and seen at the psychiatry department, 27 (27.8%) seen by a clinical psychologist, 40 (41.2%) participated in peer support clubs and 18 (18.6%) participated in family meetings.

**Table 4:** Mental health and substance use screening outcomes of adolescents screened at a paediatric HIV clinic in South Africa

	At the first screen, n (%)				Ever, n (%)			
	Age in years				Age^e^ in years			
	10 to 12	13 to 15	16 to 19	Total	10 to 12	13 to 15	16 to 19	Total
Depression^a^	501 (100.0)	339 (100.0)	247 (100.0)	1087 (100.0)	348 (100.0)	376 (100.0)	364 (100.0)	1088 (100.0)
Positive pre‐screen^b^	41 (8.2)	68 (20.1)	59 (23.9)	168 (15.5)	19 (5.5)	70 (18.6)	103 (28.3)	192 (17.6)
Positive full screen^b^	5 (1.0)	10 (3.0)	24 (9.8)	39 (3.6)	2 (0.6)	8 (2.1)	38 (10.4)	48 (4.4)
PHQ‐9^c^, mean (SD)	14.2 (5.0)	12.8 (3.4)	14.8 (4.1)	14.2 (4.0)	17.1 (8.6)	14.9 (4.0)	14.9 (4.2)	15.0 (4.2)
Suicidality^a^	498 (100.0)	328 (100.0)	241 (100.0)	1067 (100.0)	348 (100.0)	375 (100.0)	364 (100.0)	1087 (100.0)
Positive full screen^b^	2 (0.4)	8 (2.4)	14 (5.8)	24 (2.2)	2 (0.6)	8 (2.1)	19 (5.2)	29 (2.7)
Anxiety^a^	500 (100.0)	339 (100.0)	244 (100.0)	1083 (100.0)	348 (100.0)	376 (100.0)	364 (100.0)	1088 (100.0)
Positive pre‐screen^b^	33 (6.6)	67 (19.8)	64 (26.2)	164 (15.1)	16 (4.6)	66 (17.6)	105 (28.8)	187 (17.2)
Positive full screen^b^	1 (0.2)	6 (1.8)	13 (5.5)	20 (1.9)	1 (0.3)	4 (1.1)	19 (5.2)	24 (2.2)
GAD‐7^c^, mean (SD)	18.0	12.3 (1.7)	13.4 (2.5)	13.3 (2.5)	18.0	12.9 (2.6)	13.5 (3.0)	13.6 (3.0)
PTSD^a^	499 (100.0)	337 (100.0)	244 (100.0)	1080 (100.0)	347 (100.0)	376 (100.0)	364 (100.0)	1087 (100.0)
Positive pre‐screen^b^	23 (4.6)	41 (12.2)	46 (18.9)	110 (10.2)	19 (5.5)	49 (13.0)	79 (21.7)	147 (13.5)
Positive full screen^b^	5 (1.0)	7 (2.1)	12 (5.0)	24 (2.2)	5 (1.4)	9 (2.4)	24 (6.6)	38 (3.5)
PC‐PTSD‐5^c^, mean (SD)	3.6 (0.9)	3.3 (0.5)	3.6 (0.7)	3.5 (0.7)	3.6 (0.5)	3.8 (0.8)	3.5 (0.7)	3.6 (0.7)
Substance use^a^	487 (100.0)	336 (100.0)	246 (100.0)	1069 (100.0)	346 (100.0)	376 (100.0)	364 (100.0)	1086 (100.0)
Positive pre‐screen^b^	3 (0.6)	9 (2.7)	30 (12.2)	42 (3.9)	1 (0.3)	10 (2.7)	75 (20.6)	86 (7.9)
Positive full screen^b^	0 (0.0)	1 (0.3)	9 (3.7)	10 (0.9)	0 (0.0)	2 (0.5)	16 (4.4)	18 (1.7)
CAGE‐AID^c^, mean (SD)	NA	2.0	2.2 (0.4)	2.2 (0.4)	NA	3.0 (1.4)	2.3 (0.4)	2.3 (0.6)
Any condition^a^, ^d^	501 (100.0)	340 (100.0)	247 (100.0)	1088 (100.0)	348 (100.0)	376 (100.0)	364 (100.0)	1088 (100.0)
Positive pre‐screen^b^	73 (14.6)	127 (37.4)	121 (49.0)	321 (29.5)	43 (12.4)	126 (33.5)	212 (58.2)	381 (35.0)
Positive full screen^b^	10 (2.0)	22 (6.5)	43 (17.4)	75 (6.9)	7 (2.0)	21 (5.6)	69 (19.0)	97 (8.9)

Data are n (%) unless otherwise specified. For proportion, the denominator was the total population pre‐screened. Full screens were done conditionally on a positive pre‐screen. Full screen for suicidality was done conditionally on a positive pre‐screen for depression. CAGE‐AID, CAGE Adapted to Include Drugs; GAD‐7, Generalized Anxiety Disorder 7‐item scale; NA, non‐applicable; PC‐PTSD‐5, Primary Care Post‐Traumatic Stress Disorder Screen for DSM‐5; PHQ, Patient Health Questionnaire; PTSD, post‐traumatic stress disorder; SD, standard deviation.

^a^ Total population pre‐screened.

^b^ numbers and percentages of patients who screened positive at the first screening or ever in any repeated screening during the study duration.

^c^ mean (SD) test scores of adolescents who screened positive in full screens.

^d^ any condition refers to depression, suicidality, anxiety or substance use.

^e^ age was assessed at the last screen.

### 3.4 Co‐occurrence of multiple mental health and substance use problems at the first screen

**Figure 1:**
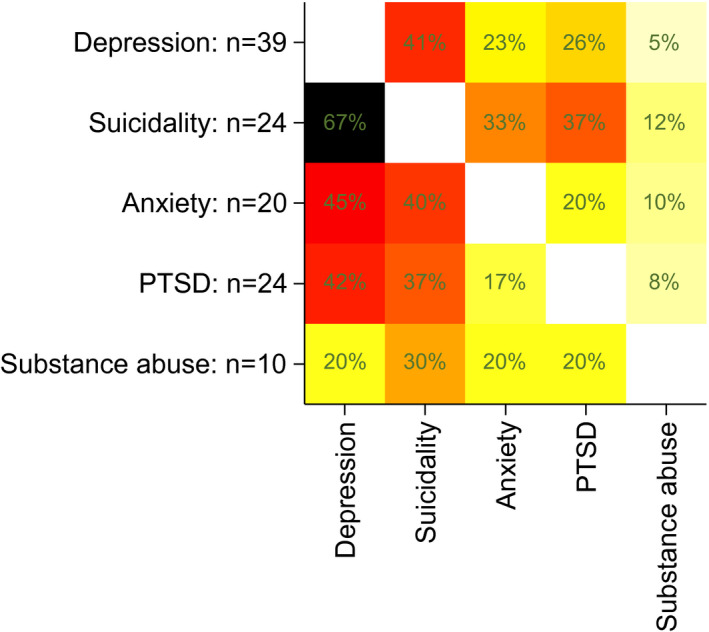
Co‐occurrence of positive full screen for mental health and substance use conditions at the first screen. Percentage of adolescents who had a positive full screen for the condition shown on the x‐axis among those who had a positive full screen for the condition shown on the y‐axis. N represents the denominator. Darker colours represent higher values. PTSD, post‐traumatic stress disorder.

Positive full screens for multiple conditions at the first screen were common: among the adolescents who had a positive full screen for one condition, 32.0% (24/75) had another positive full screen, and 18.7% (14/75) had positive full screens for two or more additional conditions. Figure 1 shows the co‐occurrence of positive full screens on the first screen for each possible pair of conditions. Positive full screens for depression and suicidality often co‐occurred: 41.0% (16/39) of adolescents who screened positive for depression also screened positive for suicidality. Other common co‐occurrences include positive depression screens among adolescents with anxiety (45.0%, 9/20) or PTSD (41.7%, 10/24).

### 3.5 Patient characteristics associated with ever screening positive in full screens

Table 5 shows aORs for patient characteristics associated with ever screening positive in full screens for mental health and substance use problems. Younger age was associated with lower odds of screening positive for all five conditions. More specifically, adolescents aged between 10 and 12 years old had 11 times lower odds of screening positive for any condition (aOR 0.09, 95% CI 0.04 to 0.19) than those aged 16 to 19 years (Table 5). There were no gender differences in the odds of positive screening for depression, suicidality, anxiety or PTSD. However, girls and young women had much lower odds of screening positive for substance abuse than boys and young men (aOR 0.19, 95% CI 0.05 to 0.66). Experience of physical violence was associated with positive full screen for suicidality, anxiety, PTSD and any condition and household conflicts were associated with positive full screen for depression, and any condition (Table 5).

**Table 5:** Patient characteristics associated with ever screening positive in full screens for mental health and substance use problems among adolescents screened at a paediatric HIV in South Africa

	Depression aOR (95 CI)	Suicidality aOR (95 CI)	Anxiety aOR (95 CI)	PTSD aOR (95 CI)	Substance use aOR (95 CI)	Any condition aOR (95 CI)
Gender						
Male					1.00	
Female					0.19 (0.05 to 0.66)	
Age at screening						
10 to 12	0.05 (0.01 to 0.21)	0.10 (0.02 to 0.41)	0.05 (0.01 to 0.37)	0.21 (0.08 to 0.56)		0.09 (0.04 to 0.19)
13 to 15	0.18 (0.08 to 0.40)	0.37 (0.16 to 0.87)	0.19 (0.06 to 0.56)	0.34 (0.16 to 0.75)	0.11 (0.02 to 0.48)	0.25 (0.15 to 0.42)
16 to 19	1.00	1.00	1.00	1.00	1.00	1.00
Experienced physical violence						
Never		1.00	1.00	1.00		
Currently or in the past		3.44 (1.55 to 7.65)	2.74 (1.09 to 6.85)	4.31 (2.17 to 8.54)		
Conflict in household						
Never	1.00					1.00
Currently or in the past	3.76 (1.97 to 7.17)					2.56 (1.53 to 4.28)

Data are adjusted odds ratios (aOR) and 95% confidence intervals (CI) for factors associated with ever screening positive in full screens for depression, suicide, anxiety, PTSD, substance abuse and any condition. PTSD, Post‐traumatic stress disorder.

### 3.6 Associations between positive full screens at the first screen and unsuppressed viral load at screening

**Figure 2:**
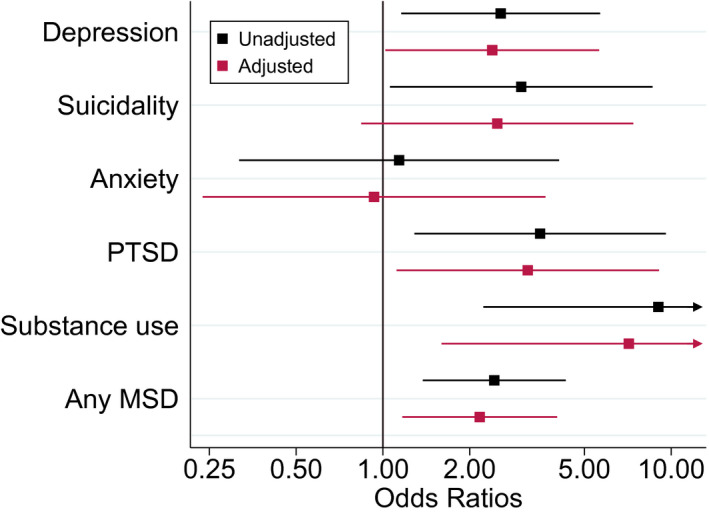
Associations between positive full screens for mental health and substance use problems at the first screen and unsuppressed viral load at screening. Adjusted and unadjusted odds ratios and 95% confidence intervals for associations between positive full screens for depression, suicidality, anxiety, PTSD, substance use and any MSD at the first screen and unsuppressed viral load (i.e. viral load above 400 copies/mL) at screening defined as the measurement taken closest to the date of screening, within 100 days before or 1 day after the screening. Odds ratios were adjusted for gender, age at screening, regimen at screening, and age, CD4 cell count and regimen at ART initiation. Odds ratios were plotted on a log scale. PTSD, post‐traumatic stress disorder; MSD, mental health, or substance use disorders.

Three‐quarters of the adolescents screened for mental health, and substance use problems (74.6%, 812/1,088) had a viral load test performed at screening (i.e. on the day of mental health screening or within 100 days before or 1 day after screening). They were included in the analysis of associations between positive mental health screens and unsuppressed viral load. The median time between viral load test and mental health screening among adolescents included in this analysis was 0 days (IQR 0 to 84 days). The prevalence of unsuppressed viral load was 17.4% (131/753) in adolescents who screened negative for all conditions, and 33.9% (20/59) in adolescents who screened positive for at least one condition. Figure 2 shows ORs and aOR for associations between positive full screens for mental health or substance use problems at the first screen and unsuppressed viral load at screening. In unadjusted analysis, adolescents who screened positive in full screens for depression (OR 2.56, 95% CI 1.16 to 5.67), suicidality (OR 3.02, 95% CI 1.06 to 8.62), PTSD (OR 3.51, 95% CI 1.29 to 9.59), substance use (OR 9.03, 95% CI 2.23 to 36.53), or any condition (OR 2.43, 95% CI 1.38 to 4.31) had higher odds of unsuppressed viral load than those who screened negative for all five conditions. In the analysis adjusted for gender, age at screening, regimen at screening and CD4 cell count, regimen and age at ART initiation, positive full screen for depression (aOR 2.39, 95% CI 1.02 to 5.62), PTSD (aOR 3.18, 95% CI 1.11 to 9.07), substance use (aOR 7.13, 95% CI 1.60 to 31.86) or any condition (aOR 2.17, 95% CI 1.17 to 4.02) remained associated with higher odds of unsuppressed viral load (Figure 2). Results were similar in the sensitivity analysis in which we used a threshold of 1000 copies per millilitre to define unsuppressed viral load (Figure S1). Positive screening for any condition remained associated with higher odds of unsuppressed viral load at the threshold of above 1000 copies per millilitre in unadjusted (OR 2.77, 95% CI 1.53 to 5.01) and adjusted sensitivity analysis (aOR 2.36, 95% CI 1.25 to 4.47) (Figure S1).

## 4 DISCUSSION

Overall, about 9% of adolescents screened positive for mental health or substance use problems with a much higher prevalence among adolescents aged 16 to 19 years (19%) than among those aged 10 to 12 years (2%). Depression was the most common mental health problem, with over 4% of adolescents screening positive for this condition. Only a few adolescents (<2%) screened positive for substance abuse. Many adolescents screened positive for more than one condition. Older age, the experience of physical violence, and household conflicts were associated with positive mental health screens. One‐third of adolescents who screened positive for a mental health or substance use problem had an unsuppressed viral load. A positive screen for mental health or substance use problems was strongly associated with unsuppressed viral load.

Our study has several strengths. It was conducted in a large public ART clinic in South Africa. The majority of HIV‐positive adolescents live in sub‐Saharan Africa and receive care in similar healthcare settings. Participants enrolled in our study received only routine services that are the standard of care for adolescent ART patients in South Africa. The study setting and our “naturalistic” approach contribute to the external validity of our study and make our results applicable to a large proportion of adolescents globally. Our sample size was large enough to conduct a multivariable analysis of factors associated with mental health and substance use problems. The longitudinal study design allowed for repeated mental health screening, and we could assess the period prevalence of positive mental health screens over two years.

Our results have to be interpreted in light of several limitations. We used screening tools, which showed acceptable diagnostic accuracy for identifying individuals with Diagnostic and Statistical Manual (DSM)‐defined mental disorders in Western and South African populations [44, 45, 46, 47, 49, 50, 51, 52, 61], but we could not validate screening tools for our young HIV‐positive study population. The prevalence of positive screening tests in our study was relatively low, and we possibly missed adolescents with mental health or substance use problems during the pre‐ or full screening. Screening tools were administered by clinic staff and ALHIV may have underreported substance use or symptoms of mental disorders as a result of social desirability bias [62]. Despite these limitations, strong associations between positive mental health screening tests and unsuppressed viral load demonstrate the clinical significance of positive screening tests for HIV treatment. We used data from routinely performed viral load tests to examine associations between mental health and unsuppressed viral load. One‐quarter of adolescents could not be included in this analysis because they had no recent viral load test result available. For one‐quarter of adolescents, we used a viral load result from a test that was performed about 3 months before the mental health screening. Changes in viral load occurring between viral load testing and mental health screening could distort associations between positive screens and unsuppressed viral load.

The prevalence of ALHIV with positive mental health screening outcomes that we observed in this study was lower than expected. About 10% to 20% of children and adolescents worldwide are estimated to be affected by mental disorders [63, 64]. In ALHIV, the prevalence of mental disorders is substantially higher than in their HIV‐negative peers [10, 33, 65]. For example in Malawi [66] and Nigeria [65], about 20% of ALHIV were diagnosed with depression based on structured diagnostic interviews [65], but in our study, only 4% screened positive for depression with a higher prevalence in those aged 16 to 19 years (10%). Our estimates for the prevalence of ALHIV with positive mental health screening outcomes are also low when compared with other African studies using mental health screening tools. In Johannesburg, one‐third of ALHIV (aged 16 years on average) screened positive for depression and one‐fifth reported substance use problems [67]. Another study from Johannesburg found that 27% of ALHIV (aged 16 years on average) screened positive for depression, anxiety or post‐traumatic stress disorder [14]. The prevalence of positive depression screens in ALHIV was 25% in Zambia [68], 26% in Rwanda [69], 32% in Malawi [66] and reached up to 51% in Uganda [70, 71]. Differences in the age of study populations, screening or diagnostic tools and cut‐offs mean that these estimates are not directly comparably. Nevertheless, the low prevalence of ALHIV with positive mental health screening outcomes in our study population suggests a low sensitivity of our screening model, particularly in younger adolescents.

Our study provides strong evidence for associations between positive mental health screening outcome and viral suppression. Existing evidence on these association is inconclusive. In a longitudinal study of children and adolescents living with HIV from the US and Puerto Rico, those with symptoms of anxiety had a 40% lower odds of having unsuppressed viral load (viral load >400 copies/mL). Symptoms of attention deficit hyperactivity disorder, disruptive behaviour or depression were not associated with viral load suppression [31]. In Botswana, a cohort study found higher rates of unsuppressed viral load (viral load >400 copies/mL) in children and adolescents who scored high for cognitive, emotional and behavioural problems than in those with a lower score [32]. In Windhoek, Namibia, a study found no evidence for associations between mental health screening tests and viral suppression [35]. In a study from New York City, disruptive behaviour disorder was strongly associated with unsuppressed viral load at a threshold of greater than >1000 copies per millilitre [34]. In contrast, another studies from New York found no evidence for associations between mental disorders and unsuppressed viral load [36].

This study is a first step towards integrating evidence‐based mental healthcare into routine HIV services at a paediatric public sector clinic in South Africa. Our research shows that adolescents affected by mental health problems have increased rates of unsuppressed viral load and need specific clinical attention. Poor ART adherence in adolescents affected by mental health problems is a plausible explanation for the observed excess in odds of unsuppressed viral load in adolescents with positive screening outcomes [26, 27]. However, the reverse direction of causality (i.e. psychiatric disorders secondary to HIV in people with insufficiently controlled HIV infection) is also a possible mechanism [6, 72]. Our study demonstrates that the integration of routine screening and management of mental health and substance use problems in a high‐burden public sector clinic in South Africa is feasible. The approach requires monitoring, ongoing staff training and improvement to ensure that its sensitivity is optimized over time. Our screening model facilitates entry into onsite counselling and psychosocial support services for further assessment and if needed a referral to specialized psychiatric care. Our experience shows that the response to mental health conditions requires a tailored approach, adapted to patients’ healthcare needs and locally available health services. Adolescents with more severe mental health problems require referral to specialized mental healthcare for proper diagnosis and mental health treatment. Adolescents with less severe mental health problems could often be managed within the ART clinic, and common underlying psychological stressors including bullying, conflicts in the home or food insecurity could be addressed through onsite counselling and psychosocial support services.

Further studies are needed to validate screening tools for ALHIV in low‐ and middle‐income countries [53, 54, 55] and to develop evidence‐based interventions for improving mental health and adherence in ALHIV affected by mental health problems.

## 5 CONCLUSIONS

ALHIV affected by mental health problems have increased rates of unsuppressed viral load and need specific clinical attention. The integration of routine mental health screening in paediatric ART programmes is a feasible approach for identifying and referring adolescents with mental health and adherence problems to counselling and psychosocial support services and if needed to psychiatric care.

## COMPETING INTERESTS

The authors declare no competing interests.

## AUTHORS’ CONTRIBUTIONS

AH, KT, MD and ME conceptualized and designed the study. SP developed the screening model and trained nurses and counsellors. KT and MM oversaw the implementation of the screening programme. SP, KB, GS, SS, PvG and MT assisted in the implementation, fieldwork or data collection. AH conducted the statistical analysis and wrote the first draft of the manuscript. NM assisted with data extraction and curation of the database. All authors contributed to the interpretation of data and provided critical inputs in the draft manuscript. All authors have read and approved the final manuscript.

## Supporting information


**Figure S1.** Sensitivity analysis of associations between positive full screens for mental health or substance use problems at the first screen and viral load >1000 copies/mL at screening. Adjusted and unadjusted odds ratios and 95% confidence intervals for associations between positive full screens for depression, suicidality, anxiety, PTSD, substance use and any MSD at the first screen and viral load above 1000 copies/mL at screening defined as the measurement taken closest to the date of screening, within 100 days before or 1 day after the screening. Odds ratios were adjusted for gender, age at screening, regimen at screening, and age, CD4 cell count and regimen at ART initiation. Odds ratios were plotted on a log scale. PTSD, post‐traumatic stress disorder; MSD, mental health or substance use disorders.
