# Supplementary figures and images for: Mental health, substance use and viral suppression in adolescents receiving ART at a paediatric HIV clinic in South Africa
Source: J Int AIDS Soc. 2020 Dec 7;23(12):e25644. doi: 10.1002/jia2.25644 (PMC7720277; doi:10.1002/jia2.25644)

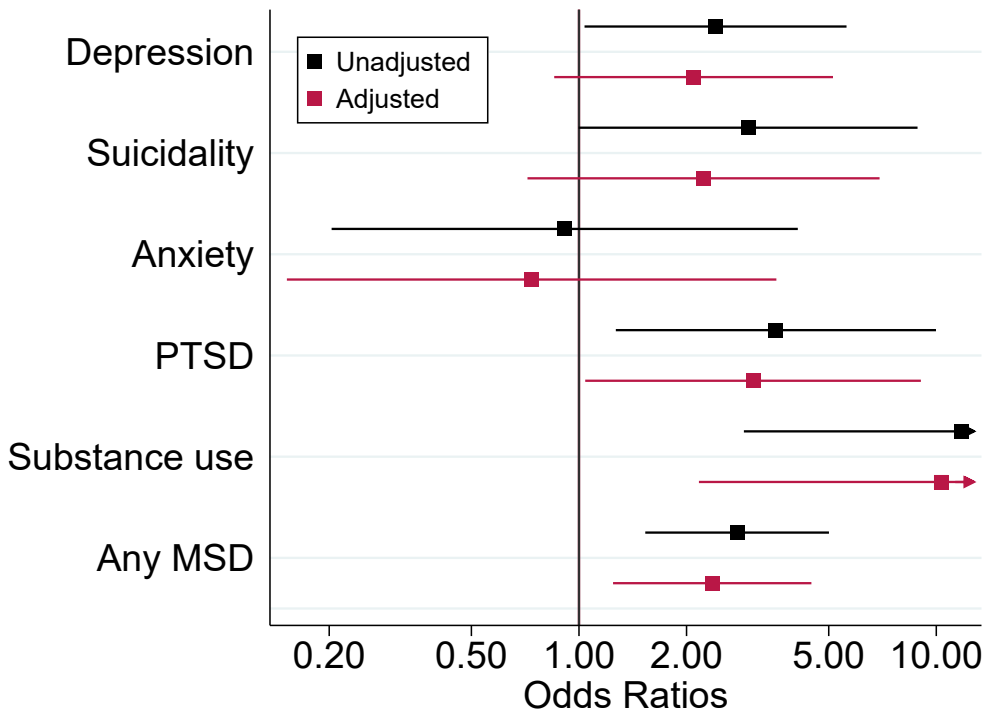

Supplement: Supplementary file 1 — Figure S1. Sensitivity analysis of associations between positive full screens for mental health or substance use problems at the first screen and viral load >1000 copies/mL at screening. Adjusted and unadjusted odds ratios and 95% confidence intervals for associations between positive full screens for depression, suicidality, anxiety, PTSD, substance use and any MSD at the first screen and viral load above 1000 copies/mL at screening defined as the measurement taken closest to the date of screening, within 100 days before or 1 day after the screening. Odds ratios were adjusted for gender, age at screening, regimen at screening, and age, CD4 cell count and regimen at ART initiation. Odds ratios were plotted on a log scale. PTSD, post‐traumatic stress disorder; MSD, mental health or substance use disorders. [file JIA2-23-e25644-s001.pdf]
